# Epigenetic Biomarkers in Cell-Free DNA and Applications in Liquid Biopsy

**DOI:** 10.3390/genes10010032

**Published:** 2019-01-09

**Authors:** Wanxia Gai, Kun Sun

**Affiliations:** 1 Department of Chemical Pathology, The Chinese University of Hong Kong, Hong Kong SAR 999077, China; wanxiagai@cuhk.edu.hk; 2 Li Ka Shing Institute of Health Sciences, The Chinese University of Hong Kong, Hong Kong SAR 999077, China

**Keywords:** plasma, cell-free DNA, cancer testing, DNA methylation, preferred ends, fragmentation pattern, tissue-of-origin analysis

## Abstract

Cell-free circulating DNA (cfDNA) in plasma has gained global interest as a diagnostic material for noninvasive prenatal testing and cancer diagnosis, or the so-called “liquid biopsy”. Recent studies have discovered a great number of valuable genetic and epigenetic biomarkers for cfDNA-based liquid biopsy. Considering that the genetic biomarkers, e.g., somatic mutations, usually vary from case to case in most cancer patients, epigenetic biomarkers that are generalizable across various samples thus possess certain advantages. In this study, we reviewed the most recent studies and advances on utilizing epigenetic biomarkers for liquid biopsies. We first reviewed more traditional methods of using tissue/cancer-specific DNA methylation biomarkers and digital PCR or sequencing technologies for cancer diagnosis, as well as tumor origin determination. In the second part, we discussed the emerging novel approaches for exploring the biological basis and clinical applications of cfDNA fragmentation patterns. We further provided our comments and points of view on the future directions on epigenetic biomarker development for cfDNA-based liquid biopsies.

## 1. Introduction

Circulating cell-free DNA (cfDNA) in human body fluids (e.g., plasma) was discovered long ago [1]. Studies found that in healthy subjects, most of the cfDNA molecules in plasma originated from the hematopoietic system [2,3]; however, in certain clinical scenarios, such as pregnancy, organ transplantation, and cancer, the related/affected tissues would release additional DNA into the plasma pool [4,5,6]. The detection of this perturbation would allow one to diagnose the abnormality of a subject in a noninvasive manner. In recent years, methods based on the analysis of cfDNA have been largely explored as an emerging technology for noninvasive prenatal testing (NIPT), organ transplantation monitoring, as well as cancer liquid biopsy [7,8,9]. For instance, the plasma DNA based fetal aneuploidy test in pregnant women was routinely deployed in more than 60 countries by 2014, and the market value is estimated to reach 3.6 billion USD in 2019 [10,11]. Studies that use plasma cfDNA for cancer testing and tumor origin determination have also demonstrated high clinical potential [2,12,13]. In these studies, a variety of approaches were developed for differentiating the cfDNA molecules released by the tissues-of-interest (e.g., circulating tumor DNA (ctDNA) in cancer patients) from the background ones, especially with the advancement of digital PCR (dPCR) and parallel sequencing technologies [14,15]. Some methods have utilized genetic biomarkers, such as the fetal-specific informative single nucleotide polymorphism (SNP) sites in pregnancies and somatic mutations in cancer patients [16,17]. However, such genetic biomarkers usually vary from case to case, which makes the development of sensitive and generalizable approaches challenging. Under this circumstance, epigenetic biomarkers are more favored. Here, we reviewed the recent studies and advances of cfDNA epigenetic markers, including DNA methylation, which is the most widely recognized epigenetic biomarker, and cfDNA fragmentation patterns, which are an emerging research direction, as well as their applications in liquid biopsy.

## 2. DNA Methylation

DNA methylation is one of the most widely studied epigenetic modifications. Most of the current studies utilized the 5-methylcytosine (5-mC) profile in the CpG context, as DNA methylation is almost exclusively found in the CpG dinucleotides [18]. The feasibilities of detecting fetal/tumor-derived DNA in the plasma of pregnant women/cancer patients using DNA methylation biomarkers were demonstrated around two decades ago [19,20]. Studies had shown that DNA methylation signatures were highly consistent between cfDNA and the genomic DNA from its tissue origins in both pregnancy and cancer models [20,21,22]. Therefore, these works opened up the possibilities of using DNA methylation patterns in NIPT and cancer diagnosis. In some aspects, the use of methylation signatures to detect fetal- or cancer-associated cfDNA in plasma has certain advantages over the methods based on genetic differences. Firstly, the DNA methylation profile is highly tissue-specific while also consistent in certain tissue type among different individuals [23]. Applying this concept in analyzing plasma DNA methylation data allows one to determine the tissue origin of cfDNA [2,24]. Such tissue-of-origin analysis allows the measurement of cfDNA from the tissues-of-interest, and thereby provides an approach to monitor the body status in a variety of pathological conditions. Secondly, compared with the genetic changes, the epigenetic modifications have higher consistency in cancer. In fact, any two tumors of the same type usually share very few somatic mutations [25], whilst the epigenetic profiles are much similar considering that they originate from the same tissue origin [2,23]. In clinical practice, informative DNA methylation biomarkers could be mined from solid tumors or the plasma DNA of cancer patients. Table 1 summarizes the recent studies utilizing such DNA biomarkers for cancer testing. Meanwhile, considering the high heterogeneity in the tumors, some studies search for biomarkers from the normal tissues of interest instead of tumors. For instance, Gai et al. discovered liver- and colon-specific DNA methylation biomarkers by mining the methylome of normal liver and colon tissues, while they were able to use these assays to separate hepatocellular carcinoma and colorectal cancer patients from healthy subjects [26]. Therefore, the work of Gai et al. suggested that tissue-specific biomarkers are reasonably consistent across cancer patients for plasma DNA-based cancer testing. Together, these studies demonstrated that a more general method could be developed by targeting informative DNA methylation biomarkers for cancer testing. Here, we reviewed the recent advances in the discovery and application of the DNA methylation biomarkers in the liquid biopsy field.

### 2.1. Tracing the Tissue Origin of Cell-Free Circulating DNA

Plasma cfDNA is a mixture of DNA released from various tissues and organs. Identifying the tissue origin of cfDNA is of great biological and potential diagnostic interest. DNA methylation patterns show the tissue-specific differences, and thus, could be used as markers for various tissues. Based on this principle, scientists have tried to utilize the tissue-specific methylation patterns to trace the tissue origin of cfDNA. Early attempts based on tissue-specific DNA methylation patterns involved detecting placenta-specific signatures in maternal plasma for NIPT. As an example, Chim et al. identified a region on the promoter of the *SERPINB5* (Serpin Family B Member 5) gene, which is hypomethylated in the placenta, while hypermethylated in the blood cells [42]. Hence, the hypomethylated cfDNA molecules associated with the promoter of the *SERPINB5* gene in maternal plasma could be determined to be fetal-derived. Subsequently, more placenta-specific biomarkers, including an exon of the *RASSF1A* (Ras Association Domain Family Member 1 isoform A) gene [43] and the promoter of the *HLCS* (Holocarboxylase synthetase) gene [44], were developed and they showed clinical utility in NIPT.

More recently, Sun et al. reported a genomewide method termed “plasma DNA tissue mapping” for tracing the tissue origin of cfDNA using methylation signatures in various tissues [2]. By comparing the plasma cfDNA methylome (using whole genome bisulfite sequencing of plasma cfDNA [22]) to a panel of reference methylomes composed of multiple tissues, the fractional contribution of each tissue to the plasma cfDNA pool could be worked out. This strategy was further validated by other groups using more complicated mathematical models [45,46,47,48]. These approaches were able to determine the relative contributions of DNA from multiple tissues simultaneously, and provided a bird’s eye view of the tissue-level composition of plasma cfDNA. Another approach reported by Lehnmman et al. relied on highly tissue-specific methylation biomarkers to identify the tissue origin of plasma cfDNA [24], which is similar to those used in the pregnancy model. They focused on detecting a few markers for one tissue at a time rather than using a genomewide approach. Other target-based studies for detecting tissue-specific cfDNA from solid tissues or hematopoietic cells were also reported [26,49,50,51]. These studies used either bisulfite targeted sequencing and/or droplet digital methylation-specific PCR (ddMSP) approaches. One major difference among these methods was that the genomewide approach was more efficient, as it provided the contributions of multiple tissues from just one experiment; while the targeted approaches were more cost-effective, and might have a wider clinical vision. Moreover, in scenarios where the increase of cfDNA concentration is contributed by more than one tissue, a method that could measure the absolute concentrations of cfDNA that originated from the tissues-of-interest would be more informative. This point was elaborated in a study by Gai et al. using metastatic colorectal cancer as a model [26]. The authors identified tissue-specific DNA methylation biomarkers for the liver and colon tissues separately, and then designed ddMSP assays to quantitatively measure these biomarkers in plasma DNA. Their results demonstrated that the absolute concentration of liver-derived DNA might perform better than the fractional concentration in terms of differentiating colorectal cancer patients, with and without liver metastasis.

### 2.2. Cell-Free Circulating DNA Methylation in Cancer Patients

In tumors, an aberrant DNA methylation pattern was often found in the promoter regions, i.e., hypermethylation of the tumor-suppressor genes and hypomethylation of proto-oncogenes [52]. Tumor-associated aberrant plasma cfDNA methylation of numerous genes was found in various types of cancers, including colorectal cancer [53], breast cancer [54], lung cancer [30,55], hepatocellular carcinoma [56], and head and neck squamous cell carcinoma [37]. These cfDNA methylation biomarkers have recently been elaborately reviewed elsewhere [57,58]. In addition, the tumors are known to be frequently suffered from global hypomethylation [59,60]. Chan et al. reported that using whole genome bisulfite sequencing of plasma cfDNA [22], this global hypomethylation was readily detectable and could serve as a general approach for the diagnosis of multiple types of cancers [61]. However, currently it is still difficult to sensitively detect this hypo-methylation signal in early cancer patients due to the low tumor DNA load in these cases.

Despite the increasing number of epigenetic biomarkers for the diagnosis of cancers, the identification of biomarkers with adequate clinical accuracy is still challenging. As shown in Table 1, although the specificity of a single marker or a panel with a few markers is relatively high, the sensitivity is usually not as satisfactory, which may be due to the noises from the background DNA. For instance, as reported in Gai et al., DNA methylation levels for the liver-specific biomarkers in the liver tissue and hematopoietic system (major source of background DNA) were 50% and 5%, respectively. However, considering that the hematopoietic system usually contributes much more plasma DNA than the liver, there could still be a high number of methylated background DNA molecules that could affect the sensitivity of the assay. With regard to this issue, recent studies had developed complex statistical algorithms and bioinformatics strategies for the analysis of cfDNA epigenetic signatures in cancer for higher sensitivities. A method reported by Xu et al. used highly selective DNA methylation biomarkers for the diagnosis and prognosis monitoring of liver cancer patients [41]. They constructed diagnostic and prognostic prediction models, with these biomarkers using a logistic regression method to improve the accuracy. Zhou and colleagues modeled the plasma cfDNA as a mixture of DNA derived from tumor and normal tissues, then they used a probabilistic model to predict tumor burden and tumor type [45,47]. Another approach by Liu et al. used targeted bisulfite sequencing to profile a list of selected CpG sites in cfDNA for cancer detection [62]. More recently, Shen et al. used an immunoprecipitation-based method to analyze the methylome of cfDNA [63]. They performed machine learning based analysis to classify multiple cancer types from the healthy controls, and they demonstrated the high sensitivity of their method which could potentially work with cancers in the early stages. These studies all involved the use of cancer-type-specific along with tissue-type-specific DNA methylation biomarkers for the cancer diagnosis and tracing the tissue origin of cancer. These cancer-type-specific biomarkers were mined by comparing the DNA methylation profiles of tumors with both the normal tissues and blood cells, and were different from the tissue-type-specific biomarkers used in works reviewed in the last section [2,24,26,46]. On the other hand, another study by Sun et al. showed that the whole fetal/placental methylome could be noninvasively reconstructed from the maternal plasma with a high accuracy and resolution [64]. If this method could be adapted to deduce the methylome of the tumor from the plasma DNA of cancer patients, such a case-specific tumor methylome would be clinically valuable in personalized therapy.

Moreover, tissue- and tumor-specific 5-hydroxymethylcytosine (5-hmC) biomarkers may also be clinically valuable in cancer diagnosis. In most human tissues, 5-hmC is not as abundant as 5-mC, while it is still detectable using current technologies. 5-hmC is also known to play roles in many cancers [65]. Recent studies have demonstrated the potential use of 5-mhC signatures in cfDNA as biomarkers for predicting the tumor type and stage [66]. In fact, the 5-hmC level in blood cells is found to be very low [67]. Considering that the blood cells are the most dominant contributors of plasma DNA, this low methylation level might therefore provide a cleaner background in plasma DNA for 5-hmC-based tissue- and tumor-biomarkers. In addition, with recent technical advances in high resolution 5-hmC profiling with low DNA input [68], we could expect that such analysis might be applied to explore the clinical potentials of 5-hmC in the near future.

## 3. Cell-Free Circulating DNA Fragmentation Patterns

Besides DNA methylation, recent studies have demonstrated the high potential of cfDNA fragmentation patterns as an emerging research direction in NIPT and cancer liquid biopsy. It had been widely recognized that cfDNA molecules were not randomly fragmented [16,69,70]; instead, they showed strong size patterns [71,72,73] which correlated well with the nucleosome footprint [74], suggesting that cfDNA fragmentation patterns may possess certain biological and/or clinical potential in liquid biopsy. However, current knowledge on cfDNA fragmentation patterns, either from a biological basis or clinical utilities, is considerably preliminary and most of the studies are still in the proof-of-principle stage. Here, we reviewed the most recently developed diagnostic approaches utilizing size, coverage, and ending patterns of cfDNA molecules in NIPT and cancer liquid biopsy. Note that most of the current studies on cfDNA fragmentation patterns are based on sequencing, as this technology could inform the exact boundary and location of each cfDNA molecule.

### 3.1. Size of Cell-Free Circulating DNA Molecules

In human plasma, studies have suggested that cfDNA molecules which originated from different tissue-of-origin may have different sizes. One well-known example is in the plasma of pregnant women (i.e., maternal plasma), where the fetally-derived DNA molecules are shorter than the maternal ones [16,72]. This phenomenon was comprehensively utilized in various applications in NIPT. For instance, Yu et al. showed that by measuring the relative abundance of short (e.g., 90–140 bp) DNA molecules to long (e.g., 160–210 bp) molecules, the authors could accurately estimate the fetal DNA fraction in the maternal plasma [75]. The fetal DNA fraction in maternal plasma is a key parameter in many NIPT applications, especially fetal aneuploidy testing [76,77]. Therefore, this work provided a novel approach that could infer this parameter directly from the plasma DNA sequencing data without external experimental requirements. In another work, Sun et al. showed that by comparing the number of reads mapped to chr21 in short and long DNA molecules, the euploid status of the fetus could be determined without the requirement of reference control subjects, and thus, it showed advantages over the conventional copy number-based methods [77,78,79]. The basis of the Sun et al. approach was that due to the size difference between the fetally- and maternally-derived DNA in the maternal plasma, the apparent fetal DNA fraction in short and long plasma DNA sequencing data subsets would be different, i.e., two mixture models of the fetally- and maternally-derived DNA were obtained through the size-based separation, thereby allowing the authors to use one subset as an internal reference to investigate the ploidy status of the fetus [78]. However, considering that the conventional NIPT methods were clinically validated using large-scale cohorts where they showed high accuracy, we think that one must be extra scrupulous when integrating the size characteristic into the analysis to take advantage of the benefits as demonstrated in the proof-of-principle studies [75,78].

Recent studies on solid organ transplantation patients [80], cancer patients [81], and mouse xenograft models [82] demonstrated that cfDNA molecules that had originated from the transplanted organ, or the tumor, were also shorter than the background DNA (which mainly came from the hematopoietic system [2,3]), suggesting the clinical potential of cfDNA size pattern in post-transplantation monitoring and cancer liquid biopsies. In fact, it had been demonstrated that when a size selection of cfDNA was employed, the detection of tumor-derived DNA in plasma could be largely facilitated [83,84]. In another study, Lam et al. utilized a size filter of cfDNA to improve the performance of early nasopharyngeal carcinoma (NPC) screening [85]. However, comprehensive studies employing this cfDNA size characteristic for cancer liquid biopsies are still limited. Considering the successful applications in NIPT, it would be of clinical significance to incorporate the size characteristic in future cancer liquid biopsy studies.

### 3.2. Coverage and Ending Pattern of Cell-Free Circulating DNA

Previous studies have suggested that cfDNA molecules were mostly generated through cell apoptosis [16,86]. The endonuclease enzymes functioning in the apoptotic DNA fragmentation procedure showed cutting preferences on the inter-nucleosomal DNA, while not that bound (or protected) by the histones [70,87], and thus, led to the coverage imbalance and specific ending patterns of cfDNA. Using a combined analysis of the cutting event and coverage pattern, Snyder et al. proposed a measurement strategy called the “Window Protection Score” to profile the nucleosome positioning from cfDNA [74]. Considering that the nucleosome positioning pattern is highly related to the cell identity [69,88], Snyder et al. further showed that they were able to trace the tissue origin of cfDNA which showed potential in deducing the tumor origin in cancer patients [74]. In another study, Ulz et al. demonstrated that different nucleosome occupancy in the promoters of expressed and silent genes resulted in differences in the plasma cfDNA coverage. Therefore, through investigating the coverage pattern of the tissue-specific promoters, one would be able to infer the tissue origin of cfDNA [89].

Another direction focuses on the cutting sites of the cfDNA molecules. Through extra-deep sequencing of cfDNA libraries generated from pregnant women, Chan et al. discovered thousands of cutting sites that showed strong usage preference by either fetally- or maternally-derived DNA, which were termed as tissue-specific “preferred ends” by the authors [90]. The same group further combined the size characteristic to mine “size-tagged preferred ends” in cfDNA and found that short and long plasma DNA molecules were associated with two set of preferred cutting sites. When located within the nucleosome structure, the authors showed that the cutting sites associated with long cfDNA molecules were mostly in the linkers, while those associated with short DNA molecules were enriched in the nucleosome cores [70]. They further demonstrated that this relative position of cutting sites was likely a universal mechanism affecting the size of cfDNA molecules, whether they came from the fetus or the mother. Hence, fetally-derived cfDNA molecules favored the cutting sites within the nucleosome cores, whilst the maternally-derived molecules favored the cutting sites in the linkers, and this preference difference may be due to the higher nucleosome accessibility in the placental cells. As a result, these works provided a systematic explanation of the cfDNA size pattern and the relative shortness of fetal DNA from the endonuclease cutting site of view. In addition, the analysis of cfDNA cutting sites also demonstrated clinical utilities, including the accurate estimation of the fetal DNA fraction and improving trisomy 21 testing [70,90,91]. Moreover, the same group also revealed the existence of liver- and tumor-associated preferred ends, which demonstrated utilities in organ transplantation monitoring, as well as cancer testing [92]. These studies suggested that the ending pattern in cfDNA molecules also reflected their tissues-of-origin and exhibited clinical potential as a novel direction in liquid biopsy, and therefore, is worthwhile for further investigations.

## 4. Discussion and Conclusions

Analysis of plasma DNA has been largely developed as an emerging technology for NIPT and cancer liquid biopsy. However, accurate and sensitive cancer detection is still challenging, especially in the early stage cancer cases, as there are lots of barriers to utilizing cfDNA for such applications. For instance, in most clinical practices, the amount of blood that could be obtained is rather limited; therefore, the usable cfDNA material is usually at a very low level. In the meantime, cfDNA is also vulnerable to contaminations by the lysed blood cells if not processed soon after blood collection. DNA isolation protocols and instruments could also yield significant variations into cfDNA [73,93,94]. However, a worldwide standardized cfDNA isolation protocol is still missing at the moment. On the other hand, the fraction of tumor-derived DNA in the plasma of cancer patients is usually very low. As estimated in a previous study [95], tumor-derived DNA fraction could only achieve a few percents in patients with relatively large tumors (e.g., 100cm^3^), while it could be lower than 0.1% in patients with smaller tumors, which is usually the case in early cancer patients. As a result, the development and validation of new methods, which may utilize genetics and/or epigenetics biomarkers, are still needed before clinical implementation for a better patientcare.

In the meantime, we also consider that there are certain limitations related to the current methods for epigenetic biomarker analyses. For instance, most of the current studies involve bisulfite conversion of the plasma DNA to utilize the DNA methylation biomarkers. However, bisulfite conversion is known to cause degradation of the input DNA [96], which results in a much lower amount of the usable cfDNA for the analysis. Furthermore, if the bisulfite converted DNA is subjected to sequencing, the abnormal GC-content (guanine-cytosine content) would introduce biases in the sequencing data [97], which may adversely affect the accuracy of the analysis. In addition, the DNA methylation profile is also known to correlate with gender, as well as ethnicity [98,99], and it also shows individual differences [100,101] and heterogeneity among tumors [102]. For instance, the PCDH10 (Protocadherin 10) gene promoter is hypermethylated in endometrioid endometrial carcinoma (EEC). However, this pattern was observable in only around half of the EEC patients investigated and the elevated DNA methylation level was also highly variable among the patients [103]. Such heterogeneity could significantly affect the performance of assays targeting the tissue/tumor-specific DNA methylation biomarkers. To this end, most of the recent cancer testing studies have incorporated a panel of multiple DNA methylation biomarkers and demonstrated diagnostic benefits (Table 1) [104,105]. On the other hand, cfDNA fragmentation pattern analysis is still in its infancy stage and current attempts could only be considered as proof-of-concept studies, considering the limited sample sizes and moderate accuracies [70,74,90,92]. In addition, the current understanding of the cfDNA fragmentation patterns is still very limited. For instance, in the preferred ends approaches, due to the high cost of extra-depth sequencing, only a few samples were explored as the discovery dataset to screen for preferred ends [70,90,92]. Therefore, it is possible that some of the identified preferred ends are only specific to the discovery dataset (i.e., patient-specific), which are not generalizable, while we currently could not differentiate them from the real informative ones. Consequently, we think that large-scale, multi-center, and comparative validity studies are needed to comprehensively investigate its performance before clinical implementation.

On the other hand, considering that DNA methylation and cfDNA fragmentation patterns are both highly informative and widely utilized in NIPT and cancer liquid biopsy, we think that it would be valuable if one could further integrate these two epigenetic biomarkers into the analysis. However, there are no such studies reported to date. One experimental method currently available is the whole genome bisulfite sequencing of plasma DNA [22]. In the protocol developed by Lun et al., the authors first ligated the sequencing adaptors to plasma DNA molecules, then they performed the bisulfite treatment, which preserved the fragment ends of the cfDNA molecules thereby potentially allowing the DNA methylation and cfDNA fragmentation pattern to be analyzed simultaneously using the same data [22]. In addition, the nanopore sequencing technology may also serve as a practical approach [106]. Previous studies have demonstrated its potential in working with plasma DNA for NIPT [107], as well as its ability to directly detect the DNA methylation profile in the sequenced DNA molecule [108]. Moreover, these epigenetic biomarkers could also be combined with the genetic ones for both cancer testing and tissue-or-origin analysis. For instance, Chan et al. have demonstrated that both the copy number aberrations and genomewide hypomethylation pattern in plasma DNA could detect patients with hepatocellular carcinoma. However, the combination of these two approaches could significantly reduce the false-positive rate for cancer screenings [61]. In another study, Sun et al. also integrated the copy number aberration context for more precise determination of the tissue origin of the tumor [2]. They analyzed the tissue contributions using DNA methylation biomarkers in the copy number gain and loss regions separately, and found that the tissues which showed increased contribution in the copy number gained regions compared to the copy number loss regions could directly inform the origin of the tumor [2]. In addition, even though it is very challenging to predict the tumor types from the somatic mutation profiles [109], previous studies have revealed that some somatic mutations are tumor-type specific, mutually exclusive, or co-occurrent in certain cancer types [110], where the information could be further combined with epigenetic biomarkers for tumor origin predictions. Therefore, we think that such integrated analysis could be expected in the near future to explore its advantages in cancer liquid biopsy.

Besides DNA methylation and cfDNA fragmentation patterns, there are other epigenetic biomarkers that have been explored for liquid biopsy. For instance, Jiang et al. found that in the plasma of hepatocellular carcinoma patients, the fractional concentration of mitochondrial DNA was significantly elevated and these DNA molecules showed a dramatically different size pattern compared to the nuclear DNA [81]. Circulating tumor cells in peripheral blood have also been discovered in many cancer types and have demonstrated high clinical potential in personalized medicine, especially in metastatic cancer patients [111,112]. On the other hand, recent studies have demonstrated that circulating cell-free RNA could also serve as valuable biomarkers for liquid biopsy [113,114,115,116]. Considering that the transcriptome analysis alone in tissue biopsies has demonstrated high performance in both cancerous status and tissue origin determinations [117], the cell-free RNA analysis is promising and deserves more research attentions in cancer liquid biopsy.

As a conclusion, epigenetic biomarkers in cfDNA provide generalizable solutions for plasma cfDNA-based NIPT and cancer liquid biopsy, and therefore, are of high research and clinical significance. We think that in the near future, highly sensitive and tissue-specific epigenetic biomarkers will be discovered for cancer liquid biopsy. With large-scale clinical trials and validations, these biomarkers could help us in improving the public health, as well as patientcare worldwide.

## Figures and Tables

**Table 1:** Summary of recent DNA methylation-based cancer liquid biopsy studies.

Study	Cancer Type ^1^	Sample Size	Marker(s)	Technical Method ^2^	Clinical Significance ^3^
Barault et al. [27]	GBM/mCRC	164/83	MGMT	Bisulfite dPCR	Indicating prognosis; predicting response to therapy
Barault et al. [28]	mCRC	182	EYA4, GRIA4, ITGA4, MAP3K14-AS1, MSC	Bisulfite dPCR	Predicting response to therapy
Chimonidou et al. [29]	BC	114	SOX17	MSP	Diagnosis (38%/98%)
Fackler et al. [30]	mBC	57	A panel of 10 markers	Multiplexed MSP	Diagnosis (91%/96%)
Gordevičius et al. [31]	PC	33	Multiple markers	Microarray	Predicting response to therapy
Henriksen et al. [32]	PC	95	A panel of 28 markers	MSP	Tumor staging
Herbst et al. [33]	mCRC	467	HPP1	MSP	Indicating prognosis; predicting response to therapy
Hulbert et al. [34]	NSCLC	150	SOX17, TAC1, HOXA7, CDO1, HOXA9, ZFP42	MSP	Diagnosis (93%/62%)
Melson et al. [35]	CRC/PC	30/30	CYCD2, HIC, VHL/ VHL, MYF3, TMS, GPC3, SRBC	MSP	Diagnosis (CRC: 83%/94%; PC: 81%/67%)
Potter et al. [36]	CRC	50	SEPT9	MSP	Diagnosis (68%/79%)
Schröck et al. [37]	HNSCC	425	SHOX2, SEPT9	MSP	Diagnosis (52%/95%); indicating prognosis
Wen et al. [38]	HCC	36	Multiple markers	MTCA-seq	Diagnosis (94%/89%)
Widschwendter et al. [39]	BC	902	EFC	BS-seq	Indicating prognosis
Widschwendter et al. [40]	OC	151	A panel of 3 markers	BS-seq	Diagnosis (41%/91%)
Xu et al. [41]	HCC	1098	Multiple markers	BS-seq	Diagnosis (83%/91%)

^1^ GBM, glioblastoma; CRC, colorectal cancer; mCRC, metastatic colorectal cancer; BC, breast cancer; mBC, metastatic breast cancer; PC, pancreatic cancer; NSCLC, non-small cell lung cancer; HNSCC, head and neck squamous cell carcinoma; HCC, hepatocellular carcinoma; OC, ovarian cancer; ^2^ MSP, methylation specific PCR; dPCR: digital PCR; MCTA-seq, methylated CpG tandems amplification and sequencing; BS-seq, bisulfite sequencing; ^3^ for diagnosis, performance is provided as sensitivity/specificity.
